# Ion Migration and Redox Reactions in Axial Heterojunction Perovskite CsPb(Br_1–*x*_Cl*x*)_3_ Nanowire Devices Revealed by Operando Nanofocused X-ray Photoelectron Spectroscopy

**DOI:** 10.1021/acsnano.4c11458

**Published:** 2024-12-11

**Authors:** Yen-Po Liu, Nils Lamers, Zhaojun Zhang, Nelia Zaiats, Anders Mikkelsen, Jesper Wallentin, Regina Dittmann, Rainer Timm

**Affiliations:** † Division of Synchrotron Radiation Research, Department of Physics, Lund University, 221 00 Lund, Sweden; ‡ Peter Grünberg Institut (PGI-7), Forschungszentrum Jülich GmbH, 52428 Jülich, Germany; § NanoLund, Lund University, 221 00 Lund, Sweden

**Keywords:** metal halide perovskite, nanowire, operando device, XPS, SPEM, ion migration, redox reaction

## Abstract

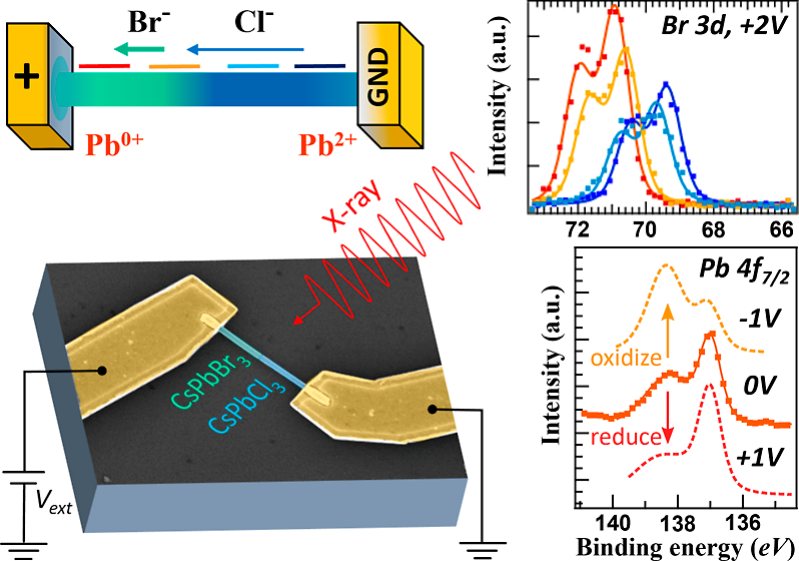

Metal-halide perovskites (MHPs) have gained substantial interest in the energy and optoelectronics field. MHPs in nanostructure forms, such as nanocrystals and nanowires (NWs), have further expanded the horizons for perovskite nanodevices in geometry and properties. A partial anion exchange within the nanostructure, creating axial heterojunctions, has significantly augmented the potential applications. However, surface degradation and halide ion migration are deteriorating device performance. Quantitative analysis of halide metal concentration and mapping of the electrical surface potential along the operating NW device are needed to better understand ion transportation, band structure, and chemical states, which have not been experimentally reported yet. This requires a characterization approach that is capable to provide surface-sensitive chemical and electrical information at the subμm scale. Here, we used operando nanofocused X-ray photoelectron spectroscopy (nano-XPS) to study CsPbBr_3_/CsPb(Br_1–*x*_Cl_*x*_)_3_ heterojunction NW devices with a spatial resolution of 120 nm. We monitored Br^–^ and Cl^–^ ion migration and comprehended the potential drop along the device during operation. Ion migration and healing of defects and vacancies are found for applied voltages of as low as 1 V. We present a model delineating band bending along the device based on precise XPS peak positions. Notably, a reversible redox reaction of Pb was observed, that reveals the interaction of migrating halide ions, vacancies, and biased metal electrodes under electrical operation. We further demonstrate how X-ray-induced surface modification can be avoided, by limiting exposure times to less than 100 ms. The results facilitate the understanding of halide ion migration in MHP nanodevices under operation.

## 1 Introduction

Metal-halide perovskites (MHPs) are investigated extensively for applications in solar cells,^1−3^ light-emitting diodes (LEDs),^4−7^ and photodetectors.^8−11^ AmCsPbX_3_ (where X = Cl, Br, or I) are particularly stable. They are popular for optoelectronics due to their eminent absorption and emission properties, along with a near-unity quantum yield in photoluminescence,^8,12,13^ covering the full visible light band spanning from CsPbI_3_ (690 nm) over CsPbBr_3_ (520 nm) to CsPbCl_3_ (410 nm).

MHP nanowires (NWs) are especially promising due to their geometry-enhanced light emission and absorption,^14−21^ and because the NW shape favors the formation of radial and axial heterostructures.^14,22−24^ Axial heterojunction perovskite NWs allow band structure engineering e.g. for multijunction solar cells, and they constrain possible ion dynamics in only one dimension. Recently, the fabrication of heterojunction MHP NW devices became possible through a controlled anion exchange process^23,25−29^ enabled by using nonpolar electron beam lithography (EBL) solvents during the synthesis.^30^

Still, a major challenge for MHP devices is their limited durability, since device performance degrades over time by exposure to moisture and light, and due to electron dynamics and ion migration.^31−34^ MHP NWs with an axial halide material junction or gradient are especially prone to undesirable anion migration, both over time and especially under applied electrical fields, which can lead to phase segregation and other detrimental effects.^31,35,36^ Thorough surface characterization is needed to understand what lies behind the degradation of the optoelectronic performance.^37−39^ X-ray photoelectron spectroscopy (XPS) can provide rich insight, revealing the oxidation state as well as electronic information such as the work function and the Fermi level.^38,40−45^ However, many traditional characterization methods are not suitable for investigating MHP nanoscale devices, including XPS, which typically has a spatial resolution of 10–50 μm or worse. Furthermore, recent studies have revealed that even MHP thin films of large spatial extension are experiencing degradation due to local inhomogeneities or grains at the few μm and sub-μm scale.^35,46,47^ Nano-focused -X-ray diffraction and X-ray fluorescence have been successfully employed to explore nanoscale structural and compositional properties of MHP thin films,^35,46,47^ and NWs.^48−51^ Nano-XPS would be an ideal tool to investigate local chemical and electronic properties of MHP NWs with high surface sensitivity and thus study ion migration along heterostructures at the relevant length scales. Moreover, combined with applied electrical bias it could gain insight into possible changes of these properties upon device operation. However, to our knowledge, no such studies have been reported yet.

In this work, we use operando nano-XPS to explore halide ion migration in single heterostructured CsPbBr_3_/CsPb(Br_1–*x*_Cl_*x*_)_3_ perovskite NW devices during electrical device operation, by mapping local elemental concentrations at the heterostructure NW surface under varying applied bias. We find that already prior to any electrical operation, diffusion of halide ions has occurred on the NW surface. By analyzing local photoelectron intensities and binding energies, we obtain the local band structure along the NW heterojunction and observe an in-built potential with local variations of several 0.1 eV in energy, probably due to charged defects and vacancies on the surface and at the material interface. Electrical field-driven ion migration across the material interface is found to occur already at an applied bias of 0.5 V and to increase with increasing bias. Upon applying 1.5 V, the potential distribution along the NW gets irreversibly changed, removing the local inhomogeneities which were observed before. These findings indicate that ion migration along MHP heterostructure devices not only modifies the local material composition, but also has the potential to heal surface defects and thus improve device performance. Furthermore, we observe a redox reaction of the Pb cations in the MHP NW upon electrical operation, which we explain by the interplay of migrating halide ions, vacancies, and the biased metal electrodes. Our findings underline both the importance of exploring and understanding surface and interface effects in MHP devices and the large potential of operando SPEM in providing insight into chemical and electronic processes taking place at the surfaces of nanoscale devices.

## 2 Results and Discussion

**Figure 1:**
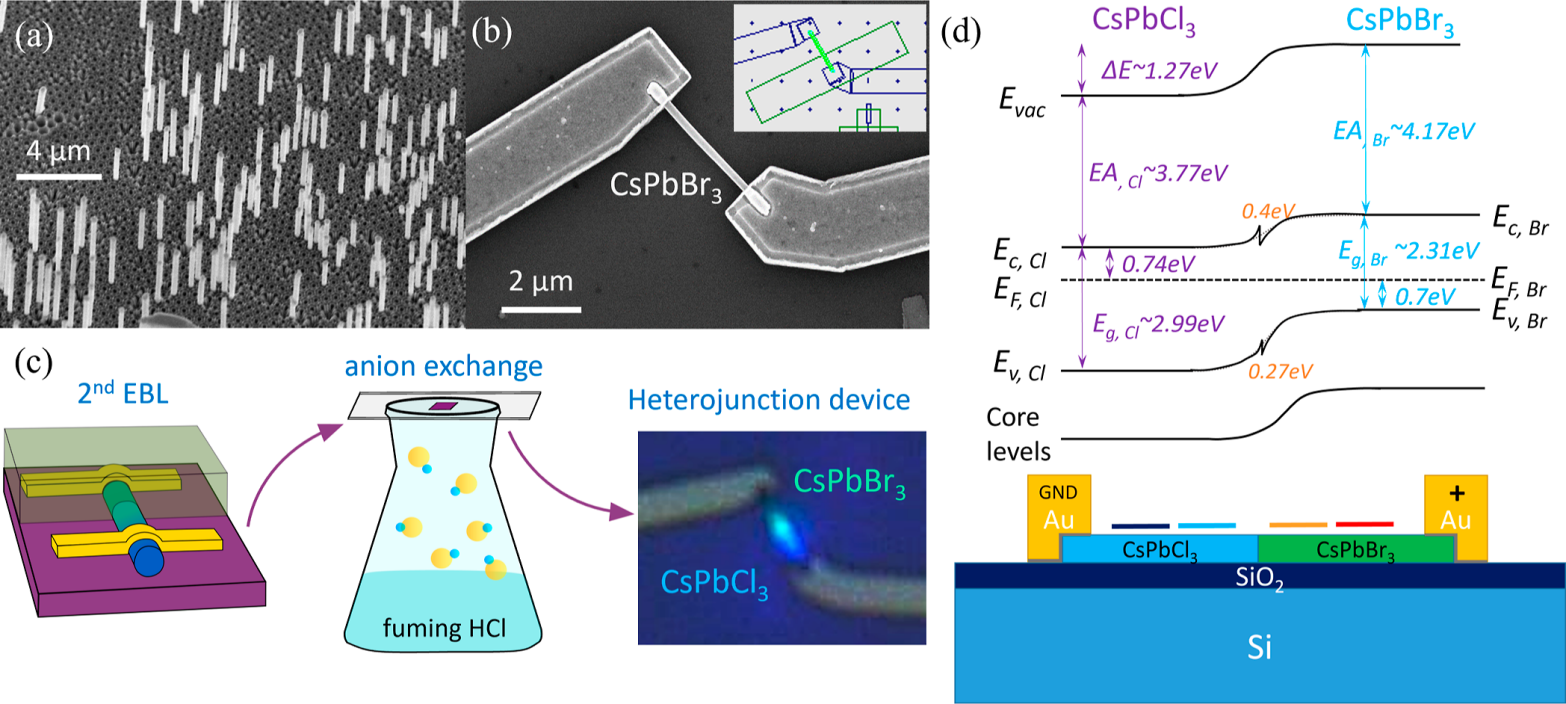
Growth, fabrication, and anion exchange of the heterojunction NW device and device band illustration: (a) SEM image showing the synthesized CsPbBr_3_ NWs with their anodized aluminum oxide template. (b) SEM image of a NW device after fabrication consisting of a NW and two electrodes. Inset: EBL layout for the second EBL for anion-exchange, showing the area being exposed to fuming HCl. (c) Illustration of the anion exchange process with EBL defining the region, fuming HCl, and a photoluminescence image taken afterward to confirm the Cl segment. (d) Expected band structure of a CsPbBr_3_/CsPbCl_3_ heterojunction device showing the band bending at the interface, which also effects core-level bands.

Heterojunction MHP NW devices have been accomplished through CsPbBr_3_ NW growth, EBL processing, partial exposure to Cl and anion exchange,^52,53^ as illustrated in Figure 1a–c. (Details for growth and fabrication can be found in the Methods section.) Individual NWs were raster-scanned through a synchrotron X-ray beam focused to a size of 120 nm while photoelectrons from a specific core-level and in a certain binding energy window were detected, a technique called scanning photoelectron microscopy (SPEM).^54,55^

The results from our MHP heterojunction NW studies are shown and discussed in six parts. We start in (Section 2.1) by evaluating the stability of the MHP surface upon intense X-ray illumination and present an approach to study the sensitive material without beam-induced modification. In (Section 2.2), this technique is first applied to a heterojunction NW deposited on a conductive Si substrate without device structure. After understanding the heterojunction NW itself, we investigate in (Section 2.3) the electrical contact quality of the electrodes to the NW, which is important for devices. A confirmed ohmic contact allows us to move on to a heterojunction NW in a device configuration in (Section 2.4). In the operando measurement presented in (Section 2.5), we follow Br^–^ and Cl^–^ ion migration by monitoring the Br 3d core-level, while applying biases of up to 2 V across the device. The migration of halide ions and vacancies can be interpreted by observing the intensity change at each position, and the potential distribution over the NW is evaluated from the energy shifts of the XPS peaks. On the double-heterojunction operando device (Section 2.6), we focus on the Pb 4f core-level under ±1 V applied over the device, which surprisingly demonstrates a redox reaction upon opposite biasing. Pb 4f core-level spectra reveal the potential gradient, the materials bonded to Pb, the status of the NW, and the defect density by the bonding state of Pb.

### 2.1 Controlling Beam-Induced Modification

The effect of beam-induced modification of a CsPbBr_3_ NW was evaluated using Pb 4f, because lead has the lowest redox-potential among the elements and this core-level is reported to be very sensitive to the environmental condition and X-ray illumination,^33,56−58^ reflecting the status of the perovskite material. We compared measurements in the conventional XPS mode, where core-level spectra from a certain nanofocus position were obtained during several seconds by measuring photoelectron intensity while varying the detected kinetic energy, and SPEM images, where the sample is raster-scanned through the X-ray beam with an exposure time at each pixel in the millisecond range while photoelectrons within a certain binding energy range are continuously detected.

**Figure 2:**
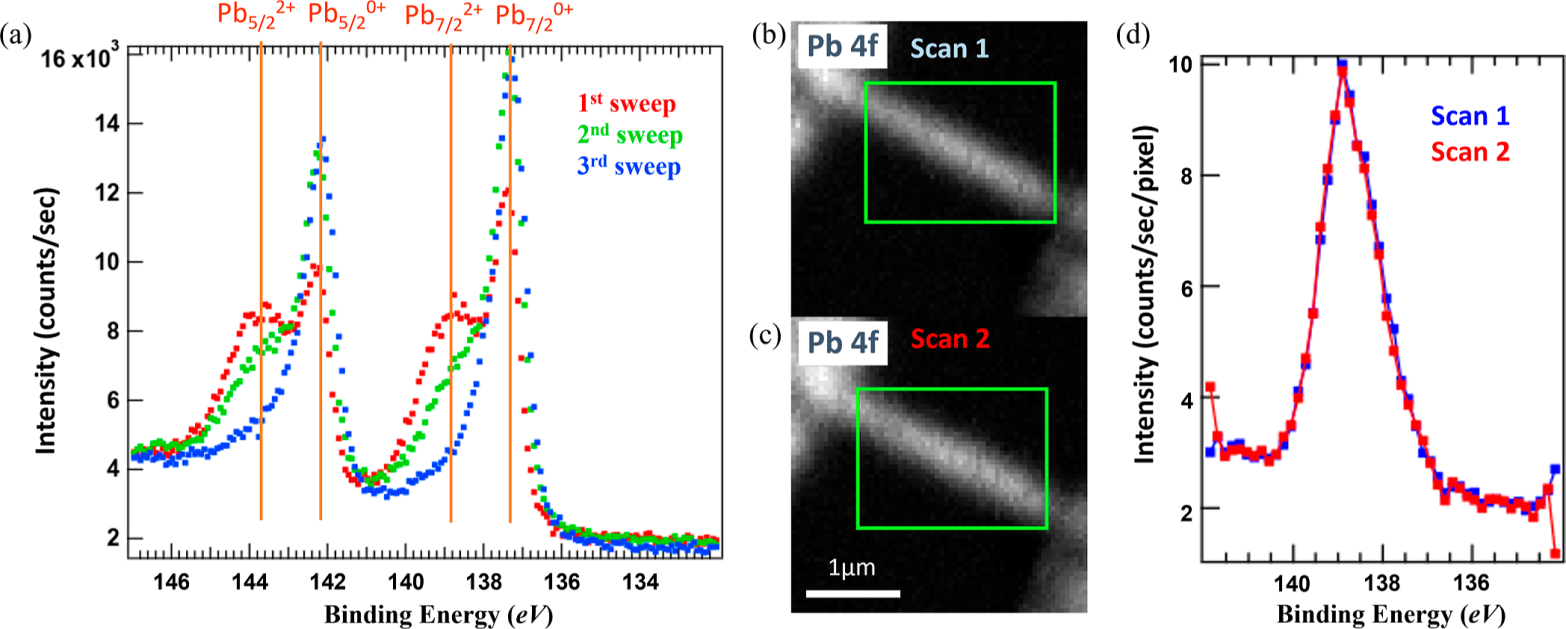
Beam modification test on a CsPbBr_3_ NW. (a) Nano-XPS with three subsequent high-resolution Pb 4f core-level spectra. (b,c) SPEM images of the Pb 4f_7/2_ core level, where the second image (c) is taken 45 min after the first one (b), with 2 other core-level SPEM images obtained in between. (d) XPS spectra extracted from the SPEM images shown in (b) (scan 1, blue) and (c) (scan 2, red) using the areas indicated by green rectangles.

In the conventional nano-XPS mode, we captured several subsequent high-resolution spectra of the Pb 4f core-level and observed a significant beam-induced modification of the NW surface upon each sweep, as shown in Figure 2a. While in the first spectrum (red), two peaks can clearly be distinguished within both parts of the doublet (4f_7/2_ and 4f_5/2_ with a spin–orbit splitting of 4.9 eV), the peaks at higher binding energies of each doublet, at 138.8 and 143.7 eV, drop in intensity at the second sweep of the spectrum (green) and almost disappear toward the third sweep (blue). The Pb 4f_7/2_ peak at a binding energy of 138.8 eV, which disappears upon subsequent scans, can be attributed to Pb in a 2+ oxidation state, which is the bulk state in CsPbBr_3_. The other peak, at a binding energy of 137.4 eV, is attributed to metallic Pb 4f_7/2_ in a 0 oxidation state. Once the device is modified by the X-ray beam or suffers from degradation, the metallic state Pb^0^ becomes dominant, which can be expressed by chemical equation: Pb^2+^ + 2Br^–^ (crystalline) ↔ Pb^0+^ + Br_2,(g)_ (degradation). Thus, we observe a severe change of the surface chemistry upon ongoing intense X-ray irradiation, including a significant degradation already upon the first XPS sweep. The observed degradation is radiation-induced, leading to the transformation of the lead halide cage into halide salts, halogen gas, and metallic lead Pb^0^. Therefore, the increased metallic lead Pb^0^ evinces higher halide vacancies. This phenomenon proves intractable to the devices, as it is intricately linked to defects within the perovskite structure.^33,59^

However, when we instead obtained SPEM images of another CsPbBr_3_ nanowire, each 50 nm by 50 nm large pixel of the image was only exposed for 75 ms (3-magnitude exposure reduced compared to the normal XPS sweep measured in Figure 2a). Subsequent images are presented in Figure 2b,c. XP spectra can be generated from the SPEM images, which are plotted in Figure 2d spatially averaged over the area indicated by green rectangles. Here, only the Pb 4f_7/2_ component is shown, due to the limited energy range of the snapshot mode. The spectra obtained from images (b) and (c) overlap almost identically, showing no change of the material upon SPEM imaging. The short exposure time and the snapshot imaging mode result in lower intensity and energy resolution as compared to the high-resolution spectra of Figure 2a, but still with appropriate signal quality. Furthermore, the spectra obtained from the SPEM images are dominated by the Pb^2+^ signal at 139 eV with an almost neglectable shoulder at the Pb^0^ energy position, which indicates that the halide ions were not decreased during the measurement.^33^

Based on this evaluation, all following results are obtained from snapshot SPEM images with short exposure times, thus avoiding beam-induced modification of the MHP material. A photon energy of 662 eV, calibrated by measuring Au 4f spectra using gold electrodes of the devices, was chosen for all images. (Details and reasons of the chosen photon energy are described in Methods.) While this approach limits the energy resolution and signal-to-noise ratio of spectra generated from snapshot images, it is of utmost importance that it avoids X-ray induced surface modification.

### 2.2 Quantitative Compositional Analysis of a Heterojunction NW

**Figure 3:**
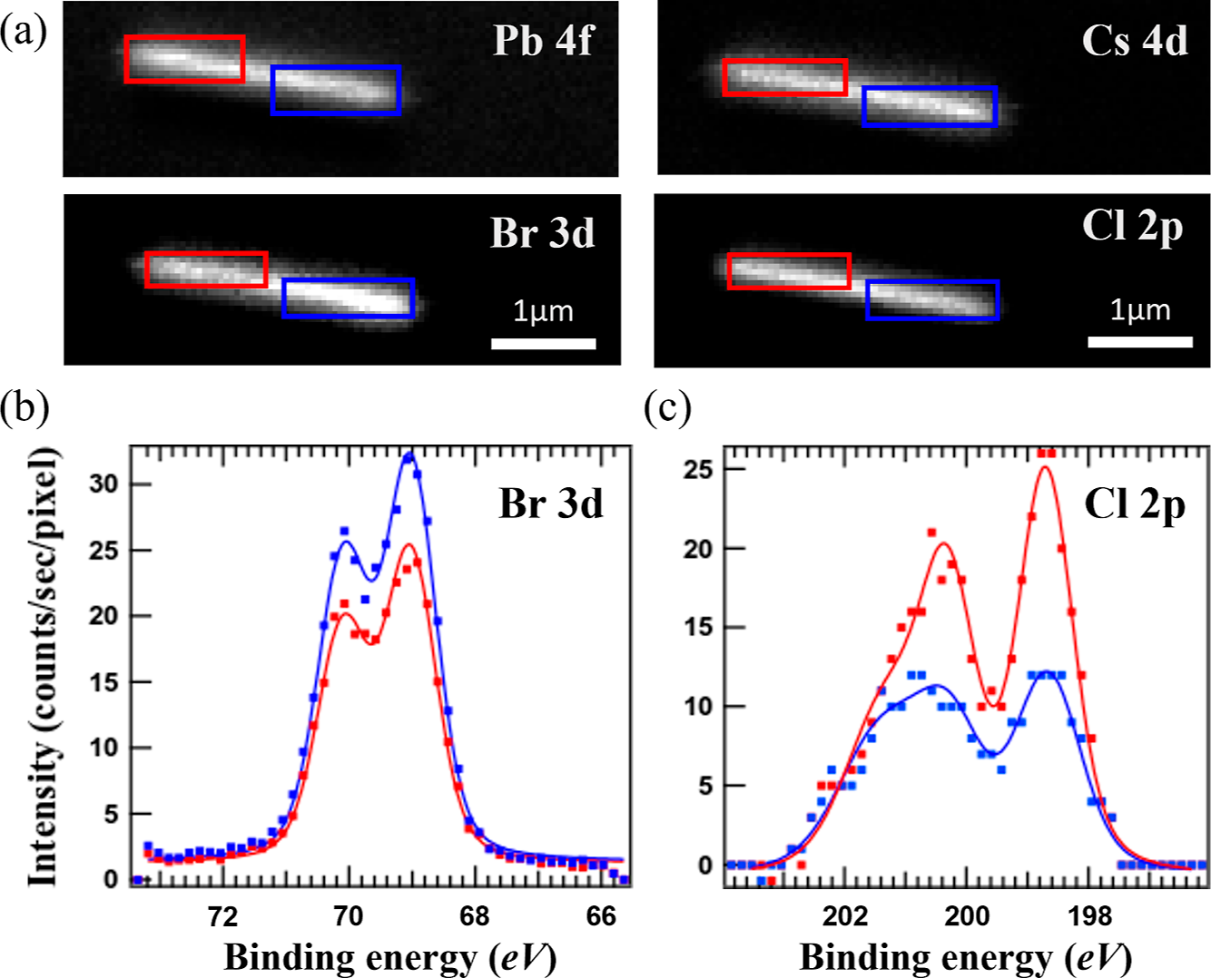
(a) SPEM images of a heterojunction NW mapping the NW element core-levels as indicated. The kinetic energy windows are fixed to 8 eV and the center binding energies are 138 eV (Pb 4f), 77 eV (Cs 4d), 69.5 eV (Br 3d), and 200 eV (Cl 2p). The blue rectangles mark the CsPbBr_3_ segment while the segment in the red rectangles has been anion exchanged with Cl. All images have the same scale. (b) Br 3d spectra and (c) Cl 2p spectra, generated from the corresponding SPEM images and spatially averaged over the areas marked blue and red, respectively.

Heterojunction NWs were synthesized from homogeneous CsPbBr_3_ NWs by local Br-to-Cl anion exchange. For this, CsPbBr_3_ NWs (growth details are presented in the Methods section) were deposited onto a Si substrate with prepatterned Au markers for positioning, followed by an EBL step which covered parts of the NWs with PMMA resist, prior to a 30 s exposure to HCl, as illustrated in Figure 1c. The segment covered with PMMA is protected from anion exchange, while the segment exposed to HCl underwent partial anion exchange, resulting in CsPbBr_3_/CsPb(Br_1–*x*_Cl_*x*_)_3_ heterojunction NWs. Once the sample was transferred to the SPEM chamber, we mapped the markers with Au 4f core-level spectra to find the specific nanowire and then imaged the core-levels of interest, including Pb 4f, Cs 4d, Br 3d, and Cl 2p. The mentioned SPEM images with a raster step size of 50 nm are shown in Figure 3a, while Br 3d and Cl 2p spectra, generated from the images at two separate segments of the heterostructure, are shown in Figure 3b,c, respectively. Different Br and Cl intensities in the two separate segments can clearly be seen.

To investigate the ratio of Cl to Br quantitatively, we first fitted the Br 3d and Cl 2p spectra at each segment with the fitting parameters described in the Methods section. After fitting, the integrated intensity (area) of the peaks were normalized with the respective XPS cross section of the core-level.^60,61^ According to the ratio of the normalized intensities of Br 3d and Cl 2p at both red and blue regions, the halide concentration of Cl in the NW surface area of the Cl-rich segment (red rectangular) is 71%, which is equivalent to 39% of the total atomic concentration.^53^ This is in excellent agreement with our previous work, where a 30 s Br-to-Cl exchange time resulted in a photoluminescence (PL) peak at a wavelength of 450 nm,^53^ which according to Vegard’s law^62^ corresponds to 38% of the total atomic concentration, further confirmed by energy-dispersive X-ray spectroscopy.

**Figure 4:**
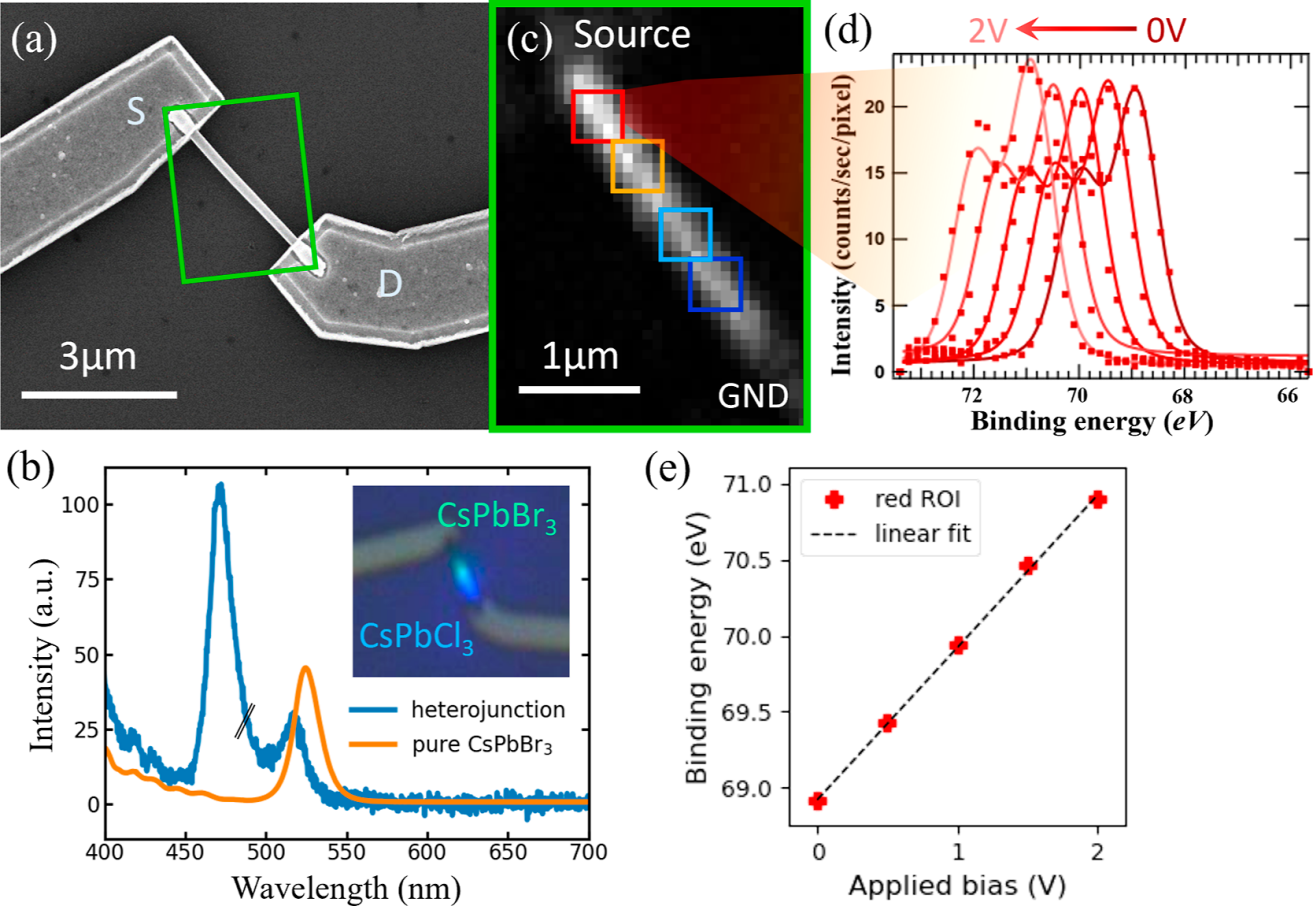
Setup and data evaluation of NW device measurements: (a) SEM image of the device after fabrication and anion-exchange; (b) PL spectrum of the heterojunction device, blue, and a pure CsPbBr_3_ NW device, orange, as reference. The blue spectrum from the device after anion-exchange showing two peaks, indicating CsPbBr_3_ and CsPb(Br_1–*x*_Cl_*x*_)_3_ segments. The intensity of the pure CsPbBr_3_ NW has been scaled down in both spectra. Inset: optical microscopy image of the area, where the PL spectrum has been acquired. The luminescence of the NW is visible. (c) SPEM image of the device at 0 V, taken at the green rectangular area marked in (a), obtained at a binding energy range of 69.5 ± 4 eV for the Br 3d core-level. The four squares (red, orange, light blue, and dark blue) are the ROIs chosen for analysis. (d) Spectra of the red ROI under externally applied bias from 0 to 2 V with a step size of 0.5 V. The red dots are raw data and the lines with gradient red colors are the fitting curves. (e) Plot of fitted Br 3d_5/2_ peak positions of (d) versus the applied bias on the source electrode. The dashed black line is a linear reference with an intercept of the fitted binding energy of the 0 V spectrum peak.

The Br segment of the NW (blue), which was not directly exposed to HCl, also shows an unexpected Cl signal, with about 31% of the halide atoms being Cl. However, keeping in mind that the majority of the XPS signal comes from a 2 nm thin surface region of the sample, the observed Cl concentration reveal only the surface situation. Considering that the anion exchange model is based on diffusion from the surface to bulk, starting with Cl ions that sit in natural vacancies at the surface, one can easily expect Cl laterally diffuse along vacancies in the surface region into the covered segment upon anion exchange. Indeed, PL from the Br-rich segment, which probes the NW core, shows a peak at 530 nm, which is close to the PL wavelength of pure CsPbBr_3_ at 520 nm (see Figure 4).This confirms that Cl diffusion into the covered area upon anion exchange, as indicated by our SPEM results, is mainly a surface effect.

### 2.3 Ohmic Contact to CsPbBr_3_ Devices

High-resistive contacts resulting in Schottky barriers are a well-known challenge for MHP-based devices.^63^ Therefore, when moving on toward operando measurements with MHP NW devices, we need to ensure that the applied bias entirely drops over the NW, and not at a Schottky barrier between the NW and the electrode. For this, we investigated a device consisting of a 4 μm long NW contacted by a source electrode and a drain electrode (5 nm Ti and 50 nm Au) on both ends, sitting on top of a SiO_2_/Si substrate, with a scanning electron microscopy (SEM) image shown in Figure 4a. After Ti/Au electrode deposition, still a 2.8 μm long part of the NW is unveiled. Among this uncovered part, a segment of 1.6 μm, defined by the dark green rectangular in Figure 1b inset, was selected for the second EBL step and exposed to HCl for anion exchange, becoming a CsPb(Br_1–*x*_Cl_*x*_)_3_ segment, called Cl segment in the later discussion. The PL spectrum and optical image from the NW, see Figure 4b and its inset, show two peaks at wavelengths of 471 nm, from the Cl anion exchanged segment, and 520 nm, from the CsPbBr_3_ segment, and two photoluminescent colors, blue and green. The source electrode is connected to the upper part, the Br segment, while the drain electrode is connected to the Cl segment. (Details of the device electrical connection for operando measurements at the beamline are shown in the Methods section.) The devices were carefully shorted with copper tape from the wire bonding until transferring the sample holder into vacuum chamber of the beamline to avoid charge spikes. Therefore, the device condition before applying any bias can be considered as pristine stage after fabrication.

**Figure 5:**
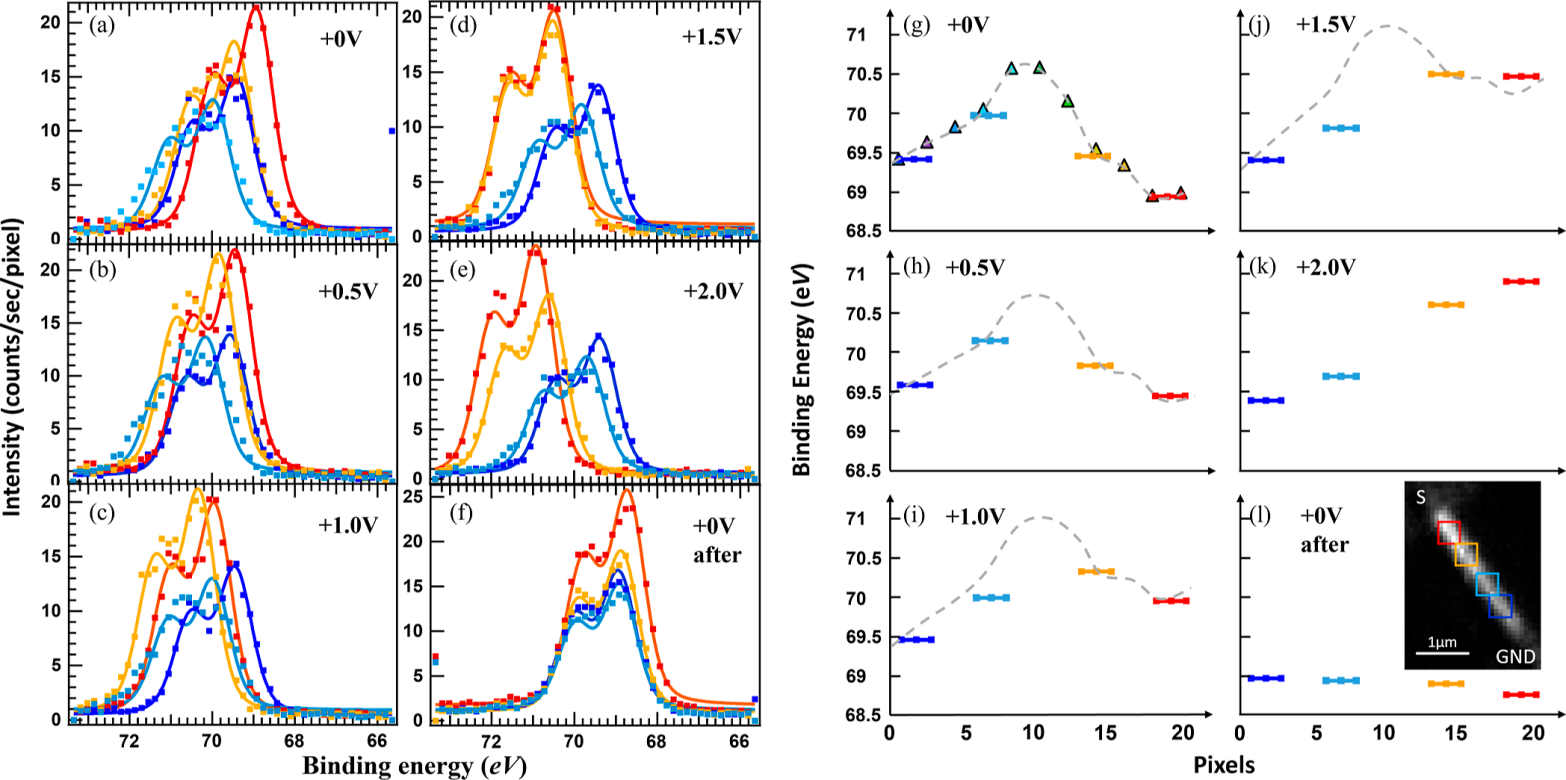
Left: XPS Br 3d spectra obtained at the four ROIs shown in Figure 4c, for different biases applied to the source electrode while the drain electrode is grounded: (a) pristine stage at 0; (b) 0.5; (c) 1.0; (d) 1.5; (e) 2.0; (f) back to 0 V after the series. Right: binding energies of the four ROIs extracted from fitting the spectra shown in (a–f). (g) The pristine stage at 0; (h) 0.5; (i) 1.0 (j) 1.5; (k) 2.0 (l) back to 0 V after the series. Inset: an SPEM image showing the four ROIs and the two electrodes. The triangles in (g) are the experimentally obtained initial in-built potential extracted from the 2 × 2 binning analysis, presented in Figure S2. The gray dashed line in (g–j) indicates the initial in-built potential overlaid by a linear drop of the externally applied bias.

For operando SPEM measurements, we scanned the NW device through the X-ray beam while a bias was applied across the NW between source and drain contacts. Here we chose a SPEM step size of 80 nm, with minimum overlapping of the beam between neighboring pixels, to reduce the overall exposure of the NW. Figure 4c shows a Br 3d SPEM image of the device, with the biased source contact on top and the grounded drain contact on the bottom. For evaluating the bias drop across the NW and the local chemical state of its surface, we selected 4 square regions of interest (ROI), colored in red, orange, light blue, and dark blue, respectively, from source to drain in Figure 4c. Each ROI has a size of 5 pixels by 5 pixels, and spectra are generated separately from each ROI, averaged over the corresponding 25 pixels (see also Figure 5). In this way, a compromise between high spatial resolution along the NW and high intensity (low noise) of the generated spectra is chosen, while still ensuring sufficiently short exposure times in order to avoid any beam damage. In Figure 4d, red and orange ROIs are located at the CsPbBr_3_ segment preserved from the photoresist, and the light blue and the dark blue ROIs at the bottom segment, which has been Cl anion exchanged.

To examine the contact quality, we analyzed the spectra of the red ROI, next to the source electrode, under different biases, as shown in Figure 4d, to observe the exact energy shifts. In the figure, five almost identical Br 3d doublets can be seen, though with a continuous shift in binding energy. These observed shifts are due to the externally applied bias, which shifts the local vacuum level, resulting in a corresponding shift of the measured binding energy of the core-level spectra. It should be noted that the Br 3d spectra are found to shift in binding energy, but do not change their peak shape upon applying an external bias, which indicates that the chemical composition and the oxidation state of the Br atoms are not affected by the external bias. Curve fitting gives a peak positions of 68.92 eV in the unbiased case (dark red curve), and a relative shift in binding energy upon the applied bias of 0.51 ± 0.05, 1.02 ± 0.03, 1.54 ± 0.03, and 1.98 ± 0.07 eV for voltages of 0.5, 1.0, 1.5, and 2.0 V applied on the source electrode (red to faded red curves), respectively. (Absolute peak positions are plotted in Figure 4e and listed in Table S1 of the Supporting Information, and the errors are from XPS fitting.) This shows that the applied bias completely drops over the NW and that an ohmic contact is formed at the CsPbBr_3_/Ti/Au interface of the device.

### 2.4 Local Potential Distribution along the Heterostructure Device

Having confirmed the formation of ohmic contacts, we are now ready to follow the local potential distribution along the heterostructured NW. Figure 5 shows Br 3d core-level spectra extracted from SPEM images, obtained at varying applied bias, at four different positions along the NW, as indicated by the four ROIs in the inset of Figure 5l, a larger image shown in Figure 4c. Already without applied bias, as plotted in Figure 5a, there is a significant shift in binding energy along the NW. The binding energy peak position is lowest for the red spectrum, in the Br-rich segment, amounting to 68.92 eV, and it increases to 69.44 and 69.96 eV for the orange and light blue spectra, before it decreases again to 69.40 eV for the dark blue spectrum, in the Cl-rich segment. The observed core-level band bending follows the alignment of the vacuum level. Accordingly, there is an in-built potential along the NW, which can be comparable to that of a pn-junction NW device.^54^

To understand this, it is necessary to consider the band structure of the heterojunction device, which is also the first step in understanding ion migration. Figure 1d shows the expected band structure for a heterojunction with pure CsPbBr_3_ and CsPbCl_3_ segments, which is plotted by Fermi level alignment. The band offsets are referenced to absolute energy levels of CsPbBr_3_ and CsPbCl_3_ from literature.^64^ According to the 471 nm peak in the PL spectrum of the investigated NW device (see Figure 4b), only 50% of the halides in the Cl-rich segment are Cl ions, and a band diagram of the device with a CsPbBr_3_ and CsPbBr_1.5_Cl_1.5_ heterojunction NW is illustrated in Figure S1c of the Supporting Information, mainly featuring smaller band offsets between the two segments resulting in an in-built potential of 0.63 eV.

The positions of the XPS peaks along a NW in a device geometry show the real local potential at the surface of the device, in contrast to a NW lying on a conductive substrate, where the entire NW is grounded by the substrate and any axial potential changes are compensated by the grounding. The experimentally observed energy positions, as shown in Figure 5a, show an overall in-built potential of about 0.5 eV, seen as the binding energy difference between both ends, red and dark blue ROIs. The observed energies confirm that the binding energy is generally lower at the Br segment (red and orange) and higher at the Cl segment (light blue and dark blue), which implies that the valence band maximum is further away from the Fermi level (more n-type) in the Cl-rich segment and closer to the Fermi level (more p-type) in the Br-rich segment, as expected. However, they do not show the expected monotonous band bending at the interface region, but include a much larger local binding energy variation, with the highest energy found close to the center of the NW. This discrepancy between expected and actually observed potential distribution could be due to Fermi level pinning at the surface, charging effects during the fabrication process, for example, upon EBL, or inhomogeneous defects within the nanowire. The opposite potential gradient in the two segments can be compared by analogy with the typical electrochemical double-layer effect.^65^ Here, we empirically evaluate the energy position of the Br 3d signal along the NW at 0 V applied bias, and with that the distribution of the in-built potential along the NW, with high spatial resolution. The procedure, making use of pairs of neighboring pixels in the SPEM images, is demonstrated in the Supporting Information (Figure S2), and the obtained potential distribution along the unbiased NW is shown by the gray dashed curve with triangle markers in Figure 5g. The core-level binding energies at the four ROIs fit well to the reference gray dashed line extracted from the high-resolution analysis in Figure S2c.

### 2.5 Br Ion Migration along the NW Device during Electrical Operation

With knowledge of the device status at 0 V, operando measurements of the NW device were performed by applying a varying bias from 0.5 to 2 V with an interval of 0.5 V. The resulting spectra for each region of interest are shown in Figure 5b–e, respectively. The binding energy peak positions of each ROI under different biases are shown in Figure 5g–l. With a bias of 0.5 V applied to the source electrode, connected to the Br segment, while keeping the opposite NW end at the Cl segment grounded, reasonable peak shifts within the NW are seen in Figure 5b. The peak positions were shifted to a higher binding energy, as shown in Figure 5h. The peak positions of the four ROIs follow extremely well the gray dashed line in Figure 5h, which shows the initially measured in-built potential distribution at 0 V (obtained as demonstrated in Figure S2) overlaid with a linear potential drop of 0.5 eV along the NW (from the red square to the dark blue one). This indicates that the applied potential of 0.5 V drops linearly over the NW and the in-built potential maintains, while it does not significantly relate to the Cl to Br ratio in the different segments. A similar behavior is observed when 1 V is applied to the source electrode (see spectra in Figure 5c) and the fitted peak positions shown in Figure 5i follow the sum (gray line) of the initial in-built potential and a linear drop of 1 eV over the NW. Keeping rising the applied bias to 1.5 V, the spectra of the red and orange ROIs, shown in Figure 5d, almost overlap in binding energy, as the electrical field due to the applied bias compensates that of the in-built potential. From Figure 5j it becomes apparent that now the voltage drops within both segments are significantly lower than that at the interface (between orange and light blue ROIs). As a consequence, the binding energy peak position of the light blue ROI, located at the Cl-rich segment close to the material interface, is lower than the overlay of in-built and externally applied bias (gray line). In other words, it rather follows a linear trend due to the applied bias and less that of the initial in-built potential. This tendency gets strongly enhanced when the bias applied to the NW device is increased to 2 V. A rather linear voltage drop over the NW, from the red to the dark blue ROIs, can be seen from the spectra in Figure 5e and the binding energy positions in Figure 5k. Hardly any indication of the initial in-built potential is left. Returning to 0 V, surprisingly, all four spectra almost overlap (see Figure 5f), and their binding energy positions (drop below 69 eV, lower than the pristine state) vary only within 0.2 eV, increasing from red to dark blue. We can conclude that the in-built potential which initially was found along the NW, probably due to charged defects at the material interface, was irreversibly removed by applying a bias of more than 1.5 V across the NW, which can be interpreted as a kind of electrical field driven self-healing of the NW. Furthermore, after biasing the device, no sharp material transition can be recognized anymore by the local potential distribution, indicating that ion migration between the two NW segments has occurred upon applying a bias of 1.5 V or more.

In addition to the peak positions, relative changes of the Br 3d intensity during device operation also indicate the occurrence of ion migration. Br^–^ and Cl^–^ ions compete for the halide ion lattice sites, meaning that an increasing (decreasing) Br 3d intensity includes a decrease (increase) of the number of Cl^–^ ions in the same surface area, especially in the segment of the NW that has undergone anion exchange. The Br 3d intensities over the whole four ROIs are plotted in Table 1. Br 3d core-levels were studied instead of Cl 2p due to the higher XPS cross-section and because Br ions are present along the entire NW. An illustration of the halide distribution over the NW prior to any applied bias is shown next to the numbers of 0 V in Table 1 (the Br segment is colored in green, while the gradient blue indicates the concentration of the Cl in CsPb(Br_1–*x*_Cl_*x*_)_3_). In the pristine state, as shown in Figure 5a and Table 1, the Br intensity is higher in the Br-rich segment (red and orange ROIs) and lower in the Cl-rich segment (light and dark blue ROIs), as expected, but with some variations: The orange ROI has a lower Br intensity than the red ROI, which indicates that Cl^–^ ions from the anion-exchanged segment have diffused along the surface, replacing the Br sites in vicinity to the interface. Similarly, the dark blue ROI shows a slightly higher Br intensity than the light blue ROI, which might be explained by Br^–^ ions from the reservoir underneath the ground electrode (protected from anion exchange) which diffused into the surface area close by. It is worth mentioning that the length of the bare NW is 2.8 μm while the Cl-rich segment is 1.6 μm long, exceeding half of the length, meaning that the actual interface is expected in the orange ROI. One should furthermore keep in mind that some amount of Cl^–^ ions is observed along the entire NW (see part II and Figure 3), telling us that even the segment without intentional anion exchange has a mixed CsPb(Br_1–*x*_Cl_*x*_)_3_ stoichiometry at the surface.

**Table 1:**
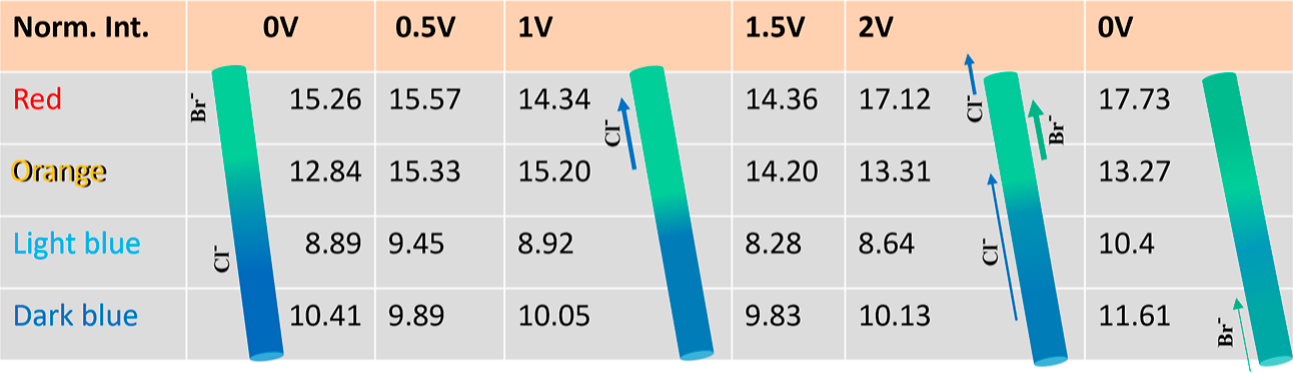
Br 3d Intensity of the Four ROIs (Red, Orange, Light Blue, Dark Blue) from 0 to 2 V Applied Bias, and Back to 0 V^a^

a Illustrating graphs are shown next to the intensities: the left-most graph shows the pristine stage. The graph at 1.0 V shows the trend of Cl^–^ migration towards the positively biased electrode. The graph at 2.0 V illustrates the further migration of the Cl^–^ and Br^–^ ions. The total amount of Br^–^ ions in the NW surface is increased after biasing (shown to the very right), as compared to the pristine stage.

When an external bias of up to 1.0 V is applied, the Br intensity decreases slightly at the red ROI (from 15.3 to 14.3), but increases significantly at the orange ROI (from 12.8 to 15.2). This indicates that Cl^–^ ions are migrating from the interface region (orange) toward the positively biased end of the NW repelling Br^–^ ions there. The smaller, more mobile, and more electronegative Cl^–^ ions, with highest electron affinity among halogens, are expected to respond stronger to an applied electrical field than the Br^–^ ions,^66,67^ resulting in Cl^–^ ion migration against the direction of the applied electrical field. The lower amount of Cl^–^ ions in the orange ROI gets compensated by an increased amount of Br^–^ ions, which may diffuse to the surface from subsurface regions as the migrating surface Cl^–^ ions leave their lattice sites. The Br intensity in the light blue and dark blue ROIs does not change significantly, which might indicate that the strong local variation of the in-built potential, which was observed at the interface, dominates over the externally applied bias.

Upon raising the bias from 1.0 to 1.5 V and further to 2.0 V, the Br intensity decreases at the orange ROI and increases at the red ROI. The ion migration trend from 1 to 2 V can be interpreted by Br^–^ ions migrating from the orange into the red region driven by the applied electric field, while the more mobile Cl^–^ ions from the red region probably have already migrated further into the very end of the NW under the source electrode, which is hidden to XPS. Cl^–^ ions from the Cl-rich segment may migrate toward the positive electrode to the orange region to fill the halide sites.

By removing the external bias, the Br distribution over the NW overall does not change significantly. Compared to the situation at 2 V, the Br intensity slightly increases at all ROIs except the orange one, which remains nearly the same. More importantly, the initial conditions (before applying any bias) are not reached again, which means that the process of bias-driven ion migration is partly irreversible. The sum of the total Br XPS signal from all four ROIs increases from 47.4 (initial 0 V) to 53.0 (0 V afterward) under the biasing series, showing that the Br^–^ ions did not evaporate from the NW surface as Br_2_, We want to point out that the synchrotron beam current varied by only 0.3% during the corresponding measurement period and that the instrumental fluctuations in X-ray intensity can be considered as less than 1%, confirming that the observed increase in signal is due to an increased amount of Br^–^ ions in the nanowire surface. This can be due either to a reduced amount of Cl^–^ ions, which have migrated to the area underneath the source electrode under applied bias and have not moved back when removing the bias, or to a reduced amount of surface vacancies, which limited the initial Br XPS signal, but became partially filled with Br^–^ ions upon device operation, similar as reported for an InAs NW device.^68^

Combining the information from the local distribution of the binding energy and from the Br intensity behavior upon applying and removing an external bias, we come to the following conclusions: charged defects at the axial NW interface (leading to a strongly local in-built potential distribution) and surface vacancies can be healed by NW device operation at about 1.5 V. On the other hand, ion migration is observed already upon 0.5 V applied bias, which turns out to be partly irreversible after device operation at 1 to 2 V.

### 2.6 Pb Redox Reaction under Device Operation

The voltage-driven migration of halide ions, on the other hand, generates vacancies. The suggested equation for CsPbBr_3_ using Kröger–Vink notation would be CsPbBr_3_ ↔ 3V_Br_^•^ + V_Pb_^″^ + V_Cs_^′^, stating that the Pb-oxidation states and intensity reveal information about the binding between Pb and halide ions and the vacancy concentration. Here, we investigate the Pb 4f core level, which can undergo significant changes between the oxidized state and the metallic state as seen above (Figure 2). When carefully avoiding beam-induced damage, as proven in part (I), the Pb states indicate binding between Pb and halide ions or vacancies and reveal the ion migration within the device.

We now move on to a double heterostructure device, which consists of a 3.7 μm long CsPbBr_3_ NW, where a 1 μm long segment in the center has been anion exchanged by HCl fuming. The NW was contacted with Ti/Au electrodes, which cover about 0.4 μm of each NW end (see Methods). The SEM image, layout for anion exchange, and the PL spectrum of the device are shown in Figure S3 of the Supporting Information. The PL spectrum in Figure S3c shows a peak at 480 nm, indicating that 36% of the halide atoms at the central Cl-rich segment are Cl. The expected band structure over the NW device is shown in Figure S4c.

**Figure 6:**
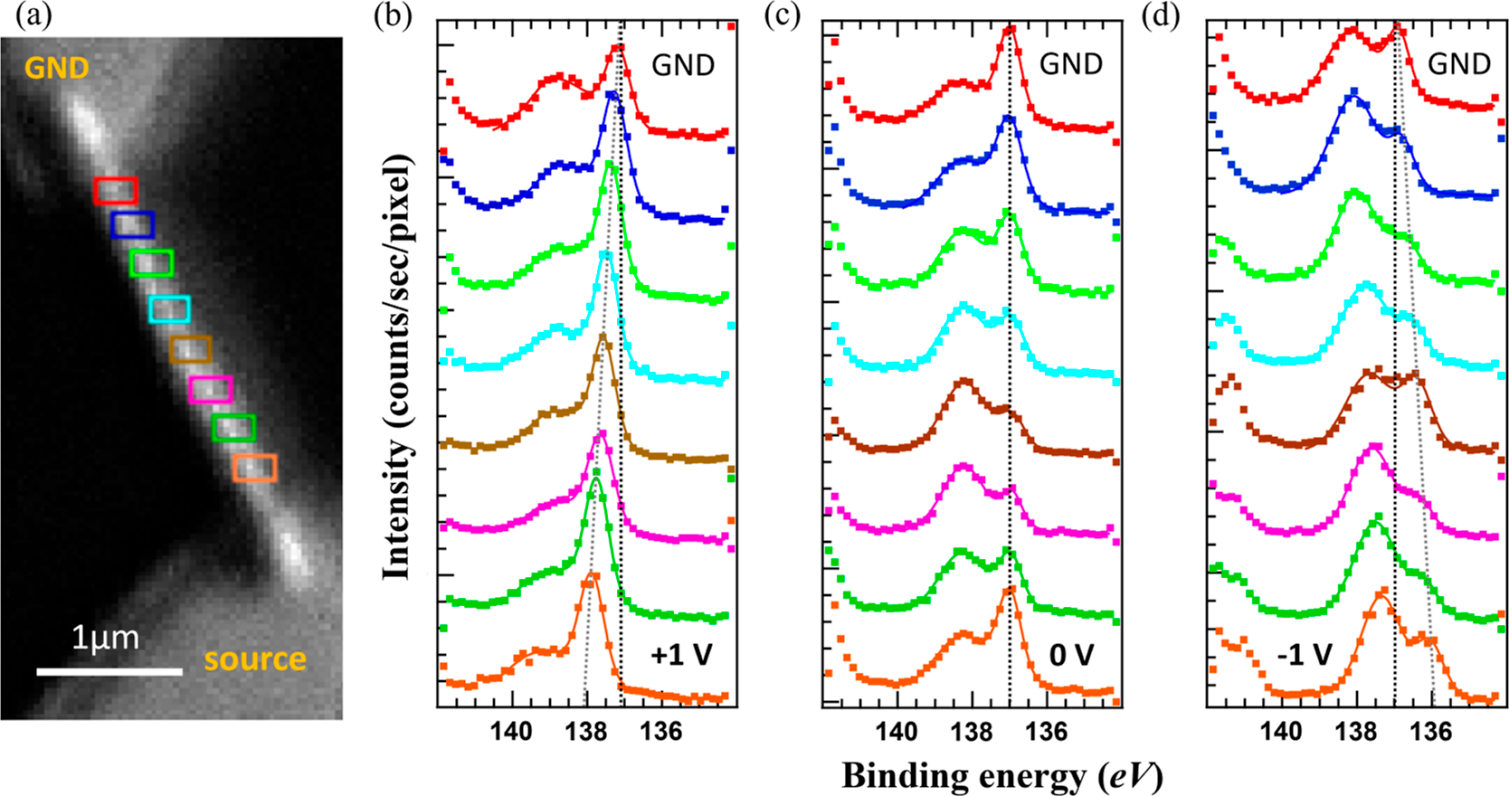
Pb 4f_7/2_ core-level data from a CsPbBr_3_/CsPbCl_3_/CsPbBr_3_ double heterostructure NW device upon operando measurements within ±1 V. (a) SPEM image, obtained at a binding energy range of 138 ± 4 eV and with an image step size of 40 nm. The positions of eight ROIs along the NW are indicated, as are the biased source electrode and the grounded (“GND”) drain electrode (b–d) Pb 4f_7/2_ spectra, extracted at the different ROIs marked in (a) from SPEM images obtained under (b) +1, (c) 0, and (d) −1 V applied to the source electrode. The black dotted lines mark the binding energy peak position of the metallic state Pb^0^ component next to the ground electrode, and the gray dotted lines indicate the trend of linear bias drop.

A Pb 4f SPEM image of the NW device is shown in Figure 6a. The device is biased with +1, 0, or −1 V applied to the bottom source electrode, and Pb 4f spectra are extracted from corresponding SPEM images at eight separate ROIs along the NW, as shown in Figure 6b–d, respectively. Each waterfall plot in Figure 6b–d shows eight spectra, from the grounded top (red ROI) to the bottom (orange ROI) of the NW. We see the Pb 4f_7/2_ peak which has two main components, the Pb^2+^ cation component at 138.5 eV binding energy (in the unbiased case) and the Pb^0^ metallic component at about 137 eV. Their ratio varies along the NW and for different applied biases. The two components can be fitted, as shown in Figure S5 of the Supporting Information. However, the transition between both states can already clearly be seen in the raw data, which are presented here.

Starting with +1 V applied to the source electrode, the spectra obtained at the eight ROIs along the NW have a rather similar shape, dominated by the metallic state Pb^0^ peak with only a small peak/shoulder at the position of the Pb^2+^ cation component. The energy position of these peaks shifts from ROI to ROI, as the applied bias drops linearly along the biased NW, as highlighted by the gray dashed line in Figure 6b. The ionic state Pb^0^ has a higher intensity at the areas closer to the anode (source) electrode, indicating higher vacancy concentrations, which agrees with the behavior of another perovskite system Fe-doped STO.^69^ The intensity at a higher binding energy side is from the tail of Pb 4f_5/2_ core level. This linear shift of the peak position along the NW also implies the absence of any relevant band bending between the three NW segments, in spite of the nominally expected in-built potential of 0.46 eV between the Br-rich and Cl-rich segments. This indicates that ion migration has already occurred due to the applied bias, leading to a more equal ion distribution along the NW. In fact, the in-built potential, symmetric at both interfaces along the NW, has been observed as a shift of the binding energy of the Pb 4f spectra for the pristine NW prior to electrical operation, as shown in Figure S4 of the Supporting Information.

After removing the external bias, the binding energy position of the Pb^0^ peak is aligned in all ROIs along the NW, as highlighted by the black dotted line in Figure 6c, and the same can be observed for the binding energy of the Pb^2+^ cation component. However, the intensity between both Pb components varies systematically along the NW: The reduced (metallic) Pb^0^ component dominates at the Br-rich segments (both ends), while the oxidized Pb^2+^ component is stronger at the Cl-rich segment (the middle part) of the NW. Once moving to a negative bias of −1 V, a linear voltage drop is observed again along the NW, now in opposite direction as for the +1 V case, as shown in Figure 6d. More importantly, now the oxidized state of the Pb^2+^ component is dominating along the entire NW, equally for the Cl-rich and Br-rich segments. In other words, the majority of the surface Pb atoms, which have been found in a reduced (metallic) state upon positive applied bias, became oxidized by removing the positive bias or applying a negative potential.

The observed behavior of reducing (oxidizing) the surface Pb atoms by applying a positive (negative) bias should be considered as a redox reaction upon device operation. Furthermore, the redox reaction is slightly different for the different segments of the NW, since in the Cl-rich segment a removal of the positive bias is sufficient for reoxidizing the Pb atoms, while in the Br-rich segments an applied negative bias is required. Generally, a redox reaction includes two partners, where one material is reduced while the other one is oxidized. In our metal halide perovskite NWs, Cs and Pb are typically considered as cations, and Br and Cl as anions. Accordingly, the oxidized +2 state is considered as bulk state for Pb, while the metallic 0 state comprises the reduced case, which requires extra electrons to be provided to the Pb atoms. Two cases shall be discussed:

First, one might consider that Br^–^ or Cl^–^ ions will be reduced in a redox reaction with Pb, providing the extra electrons. However, the very high electronegativity of Br and especially Cl speaks against this option. Indeed, when we look at Br 3d spectra of the Br-rich NW segments which were obtained at different applied biases, in parallel with the Pb 4f spectra of Figure 6, we always observe the same line shape and a peak binding energy position which only shifts due to the applied bias, as shown in Figure S6 of the Supporting Information. This confirms that the oxidation state of the Br ions is not changed here upon biasing. However, in the case of +1 V applied bias, Br 3d spectra of the Cl-rich NW segment show a different shape and are observed at slightly higher binding energy than expected from a linear drop of the applied bias, see Figure S6b. This indicates that some of the Br ions might change into a less negative oxidation state under positive bias if there are Cl ions (with an even higher electronegativity) around. A partial oxidation of the Br ions only in the Cl-rich segment can explain why a different reduction behavior of Pb is observed in this area, as compared to the Br^–^rich segments.

Second, we need to consider the interface between the perovskite material and the metal electrode. Zhao et al. reported that the deposition of an Al electrode onto a CsPbBr_3_ thin film leads to a spontaneous reduction of the Pb together with the oxidation of the electrode material, even without an applied bias.^57^ The situation is different here, where we obtain SPEM data from a bare NW which is contacted by Ti electrodes at its ends. Still, a combination of halide ion (and vacancy) migration with oxidation or reduction of the electrode material can possibly explain the observed redox behavior. Here, we propose that Br^–^ or Cl^–^ ions that migrate toward the biased electrode, leaving behind Br and Cl vacancies, can diffuse into the metal electrodes and react with the Ti, similar to the oxygen exchange in the SrTiO_3_ case.^70^ We see evidence for such a behavior when we compare the Pb 4f spectra obtained at the two Br-rich NW segments for +1 and −1 V applied bias (see Figure 6b,d): even though the material heterostructure is symmetric along the NW, with the Cl-rich segment in the center, the Pb 4f_7/2_ intensity ratio between the oxidized and the metallic components is strongly asymmetric, even if we consider an applied potential of opposite bias. In the NW segment close to the more positively biased electrode (source electrode at +1 V, grounded electrode at −1 V), we find more of the Pb atoms in the metallic state, while there are more Pb atoms in the cation state close to the more negatively biased electrode (ground at +1 V, source at −1 V) – probably because the Br^–^ ions are trapped in the positively charged Ti electrode, leaving behind Br vacancies in the NW which can reduce the Pb atoms, while a negatively charged Ti electrode pushes back Br^–^ ions into the NW, where the Pb atoms get reoxidized. The Kröger–Vink notation next to the source electrode wouldbe V_Cs_^′^ + V_Pb_^″^ + 3V_Br_^•^ (at + 1 V) ↔ CsPbBr_3_ (at – 1 V). This supports the viewpoint of section (v) that halide ions can be stored in and underneath the electrodes under bias, and being released back to the NW between the electrodes upon no or opposite bias.

## 3 Conclusion

We demonstrated a pioneering operando nano-XPS study of a heterojunction metal halide perovskite NW device. Br^–^ and Cl^–^ ion migration in perovskite NWs under electrical device operation, as well as a redox reaction of the Pb ions, are revealed by monitoring Br 3d and Pb 4f core-levels using operando nano-XPS. Quantitative measurement by SPEM reveals local ion concentrations and indicates vacancies, surface defects, and influence of the metal contacts, making full use of the high surface sensitivity of XPS. Nano-XPS expands possibilities for studying subμm NW devices, but it can similarly be used for exploring local surface chemical and electronic properties of thin film MHP devices.

Potential mapping over the NW devices with ohmic contacts shows a locally varying in-built potential along the surface of the NW device in pristine status, probably due to local surface defects. These local inhomogeneities get removed upon applying an external bias of 1.5 V, which instead drops linearly over the NW. From the in situ mapping of the local surface potential and Br intensity variations, together with the proposed band structure, the process of defect removal and halide ion migration can be explained. Furthermore, these observations indicate that the more electronegative Cl^–^ ions have an even higher mobility within the NW than the Br^–^ ions. Ion migration occurs already on unbiased heterostructure perovskite NWs, but gets enhanced under electrical operation. Removal of defects and vacancies on the perovskite NW surface are observed for applied biases as small as 1.0 V.

Our results further indicate a significant impact of the contact electrodes on the ion migration and perovskite NW surface chemistry. First, the NW material underneath the metal electrodes plays a role as halide reservoir, possibly enhancing chemical reactions of the halide ions with the electrode material. Second, from the Pb 4f spectra we observe a clear redox reaction at the NW surface upon biasing in opposite polarization, which we explain by an interplay of Pb and halide ions and vacancies in the NW and metal atoms in the electrodes. The reversible oxidation state Pb^2+^ conforms with the self-repair of surface vacancies upon biasing, and the slightly different redox behavior of Br-rich and Cl-rich segments confirms the higher mobility of Cl^–^ ions. For future perovskite devices, it is important to include this redox reaction, mobilities of ion migration, and electrode impacts in modeling to improve device design for durability and performance.

The nano-XPS method, performed under well-controlled conditions circumventing X-ray induced perovskite degradation, gives the opportunity to comprehend the local chemical composition, relative concentration and bonding states of the elements, and local potential mapping. The method is not restricted to MHP NWs, but our results demonstrate how nano-XPS and especially SPEM can reveal local composition and dynamic behavior of sensitive or partially instable nanostructure devices even under operando conditions, understanding physical and chemical effects at the nanoscale that can limit or enhance nanodevice performance.

## 4 Methods

### 4.1 Nanowire Synthesis, Device Fabrication, and Anion Exchange for Heterojunction MHP NW Devices

The CsPbBr_3_ perovskite NWs were solution synthesized in anodized aluminum oxide (AAO) templates, constraining the form of diameters between 200 and 300 nm and a length of 1–4 μm, as shown in Figure 1a, using the same recipe as in previous work.^30,53^ The grown NWs were then mechanically transferred using an edge of cleanroom tissue from the AAO surface to a Si (for bare NW deposition) or SiO_2_/Si (for fabricating devices) substrate with prepatterned markers for locating the randomly deposited NWs by optical or scanning electron microscopy. A follow-up MHP compatible electron beam lithography (EBL) process using poly(methyl methacrylate) (PMMA) and an o-Xylene/hexane-based developer is used for defining the electrodes, and then 20 nm Ti and 200 nm Au are deposited to contact both ends of the NWs, as source and drain. A correspondingly fabricated NW device is shown in Figure 1b. Still, a heterojunction device needs to be processed, via a gas-phase anion exchange process using HCl:^52,53^ A second EBL process is performed to define the area to be anion exchanged with Cl. After the developing step and with the photoresist still on, the sample was taped on a carrier facing down to a 5 mL solution of 37% aqueous HCl in a beaker at 24 °C for 30 s, creating the CsPb(Br_1–*x*_Cl_*x*_)_3_ segment, called Cl-rich segment in the later discussions. The anion exchange process is illustrated in Figure 1c.^53^

### 4.2 Nano-XPS and SPEM

Nano-XPS and SPEM were carried out with high spatial resolution at the ESCA Microscopy beamline of the ELETTRA synchrotron facility, Italy, using a focused X-ray beam spot of down to 80 nm × 120 nm size. Nano-XPS in the conventional XPS mode sweeps the photon energy in the desired energy range with any reasonable energy steps, and the detector measures the photoelectron kinetic energy to get XP spectra in a specific point, as large as the beam size. On the other hand, in the snapshot mode and SPEM, a fixed photon energy is used, and the XPS electron detector resolves the kinetic energy by the energy channels, where the energy resolution is limited to the channel numbers of the detector and the binding energy window is defined by the selected pass energy.^54,71^ At the ESCA Microscopy beamline, the detector comprises 48 energy channels, which can be read out simultaneously for obtaining SPEM images and nano-XP spectra in snapshot mode. In our measurements, the photon energy is chosen as 662 eV, allowing to obtain Cs 4d, Pb 4f, Br 3d, Cl 2p, O 1s, C 1s, and Au 4f core-levels while avoiding overlap with oxygen Auger electrons, as shown by a survey scan in Figure S1b of the Supporting Information. All spectra have been calibrated by the Au 4f core level measured at a grounded electrode, assuming a literature Au 4f_7/2_ binding energy of 84.1 eV.

### 4.3 Electrical Connections for Operando Measurements

There are four electrical channels into the UHV chamber at the beamline, and each channel contacts a specific spring on the sample holder carrier once it is inserted. The springs (mounted on the screws on the front side) are connected by Cu wires to the corresponding pin of a 14-pin commercial female sample plug (black), as shown in Figure S1a. The male 14-pin commercial ceramic sample plug has golden pads for wire-bonding, and a rectangular area in the middle to mount the sample. The wire-bonding can then electrically connect device electrodes of the sample to the electronics at the beamline.

### 4.4 XPS Fitting Parameters

All spectra are fitted using the Igor Pro 7 software, assuming a linear background and a Voigt function for the peak shape. For the Br 3d (Cl 2p, Pb 4f) core levels, a Gaussian full width of half-maximum (fwhm) of 0.8 eV (0.85–1.05, 0.8–1.0 eV), Lorentzian fwhm of 0.28 eV (0.4, 0.3 eV), branching ratio of 0.67 (0.5, 0.75), and spin-splitting of 1.08 eV (1.6, 4.82 eV) were used. In the Cl 2p core level spectra, an additional singlet with a binding energy of 2.7 eV above that of the 2p_1/2_ peak is needed for successfully fitting the shoulder on the high binding energy-side. The energy shift between the oxidation state and the metallic state of Pb 4f is 1.35 eV.

## Supporting Information Available

Photograph of the operando sample, expected band structure, and XP survey spectra (Figure S1); Br 3d core-level mapping along the biased heterojunction device with higher spatial resolution (Figure S2); energy shifts upon operando biasing (Table S3); SEM picture and PL spectrum of the double heterostructure NW device in pristine stage (Figure S3); pristine stage Pb 4f spectrum of the double heterostructure NW device (Figure S4); fitted components of the Pb 4f_7/2_ core-level spectra along the double heterostructure NW (Figure S5); Br 3d core-level data from the double heterostructure NW device measured in parallel with the Pb 4f data which are shown in Figure 6 (Figure S6) (PDF)

## Abbreviations

MHP: metal-halide perovskite
NW: nanowire
SPEM: scanning photoelectron microscopy
STEM: scanning transmission electron microscopy
XPS: X-ray photoelectron spectroscopy
