# Supplementary material for: Ion Migration and Redox Reactions in Axial Heterojunction Perovskite CsPb(Br1–xClx)3 Nanowire Devices Revealed by Operando Nanofocused X-ray Photoelectron Spectroscopy
Source: ACS Nano. 2024 Dec 11;18(51):34763–75. doi: 10.1021/acsnano.4c11458 (PMC11673567; doi:10.1021/acsnano.4c11458)
Supplement: Supplementary file 1 — nn4c11458_si_001.pdf [file nn4c11458_si_001.pdf]

Supporting information

Ion Migration and Redox Reactions in Axial Heterojunction Perovskite $CsPb(Br_{1-x}Cl_x)_3$ Nanowire Devices revealed by Operando Nano-focused X-ray Photoelectron Spectroscopy

*Yen-Po Liu[*1,2,3], Nils Lamers[1,3], Zhaojun Zhang[1,3], Nelia Zaiats[1,3], Anders Mikkelsen[1,3], Jesper Wallentin[1,3], Regina Dittmann[2,3], Rainer Timm[1,3*]*

[1] Division of Synchrotron Radiation Research, Department of Physics, Lund University, 221 00 Lund, Sweden

[2] Peter Grünberg Institut (PGI-7), Forschungszentrum Jülich GmbH, 52428 Jülich, Germany

[3] NanoLund, Lund University, 221 00 Lund, Sweden

Table of contents

Figure S1 – A photo of the sample holder, XPS survey spectrum, and band structure of the $CsPbBr_3/CsPbBr_{1.5}Cl_{1.5}$ heterojunction NW.

Figure S2 – SPEM core-level mapping with 2 pixel x 2 pixel binning.

Table S1 – Peak fitting positions with biased source electrode at the red ROI.

Figure S3 – Information of the double heterostructure NW device: SEM image, EBL layout and PL spectrum

Figure S4 – Pb 4f spectra and the band structure along the pristine stage double-heterojunction NW device

Figure S5 – Pb $4f_{7/2}$ spectra fitting of metallic state and oxidation state along the NW device

Figure S6 – Br 3d core-level data from the double heterostructure NW device

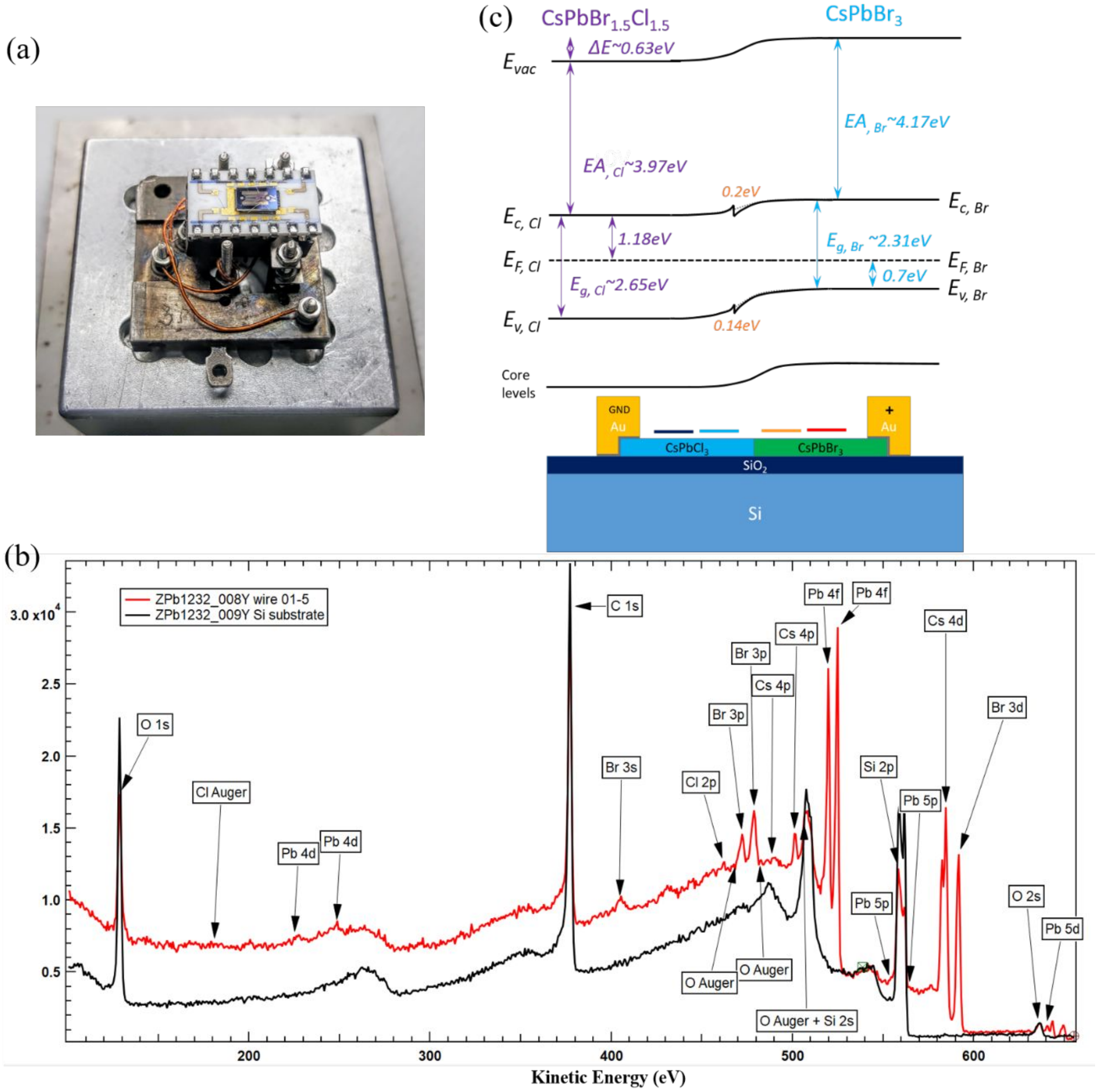

Figure S1. (a) A photo of the sample holder showing the spring connected Cu wires, a ceramic chip carrier with gold pads, and a wire-bonded sample. (b) Survey spectra taken at the position of a MHP NW (red) and at the bare Si substrate (black), for comparison. No Auger peaks are overlapping with the relevant core-levels. (c) Band structure of the $CsPbBr_{1.5}Cl_{1.5}$ heterojunction NW device showing the band bending at the interface and the expected core-level band.

We analyze and map the core-level band behavior at high resolution using binning of only 2 pixels by 2 pixels, in total 11 squares over the device, at 0 V. The eleven spectra are shown in Figure S2 (b), extracted from the eleven ROIs marked in Figure S2 (a). After fitting the eleven Br 3d spectra, a chart showing the peak binding energy along the NW is presented in Figure S2 (c).

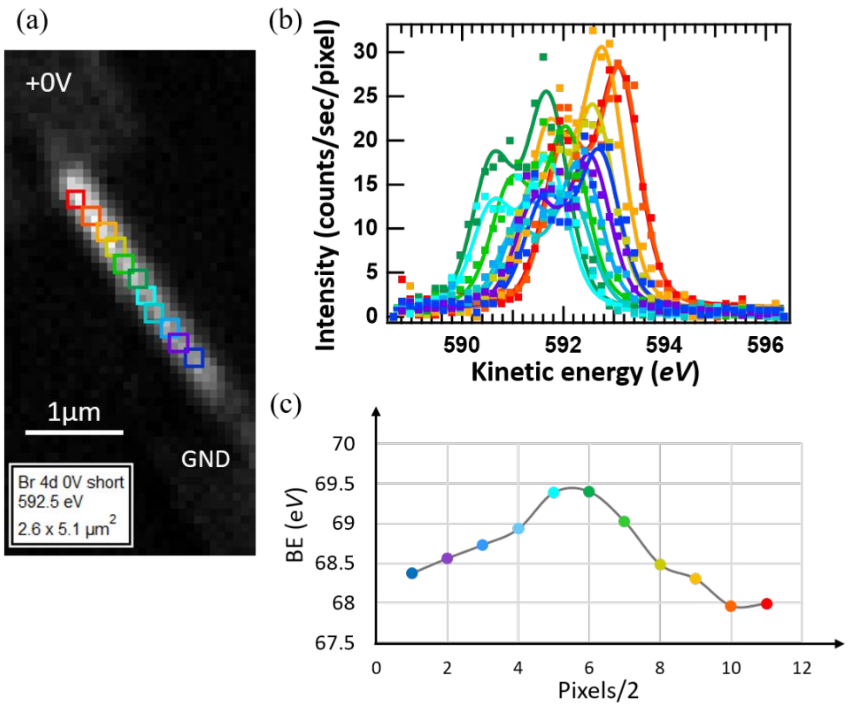

The grey curve indicates the in-built potential within the NW device.

Figure S2. Core-level mapping with 2 pixel x 2 pixel binning: (a) SPEM image at the energy of the Br 3d core level of the device. The colored eleven ROIs along the NW give a detailed information about the pristine status of the device. (b) The eleven spectra extracted from the eleven ROIs. The spectra are extracted from the square marked with the same color. (c) Peak positions of the fitted 11 spectra.

Area Red	Peak position
0V	68.92
0.5V	69.43 (+0.51)
1V	69.94 (+1.02)
1.5V	70.46 (+1.54)
2V	70.90 (+1.98)

Table S1. Peak positions at the red region in Figure 4(d) while the source electrode is biased. The numbers in the parenthesis show the difference to the peak position at 0 V.

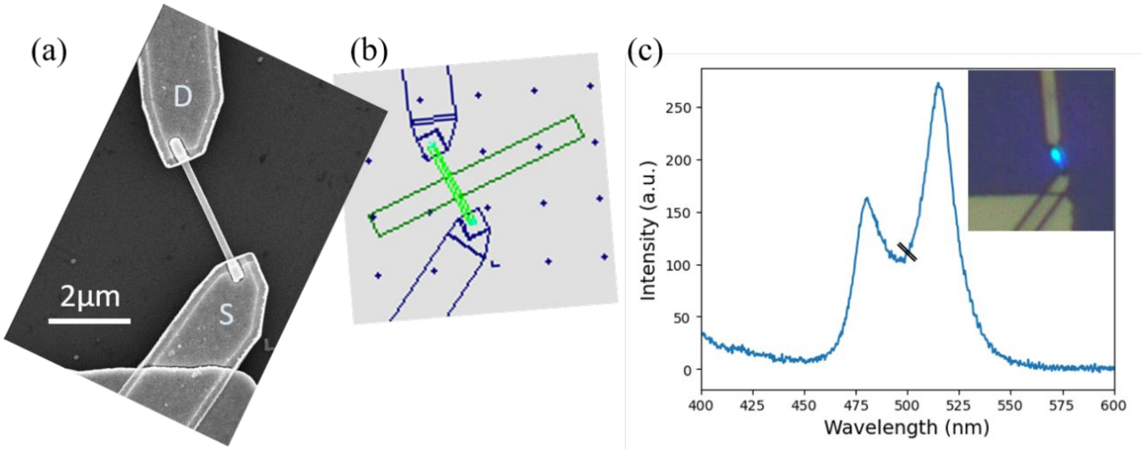

Figure S3. The double heterostructure NW device: (a) SEM image after fabrication and anion-exchange; (b) EBL layout for the anion-exchange showing the area being exposed to HCl fume; (c) PL spectrum of the device after anion-exchange showing two peaks, indicating $CsPbBr_3$ and $CsPb(Br_{1-x}Cl_x)_3$ segments. The intensity of the nominally pure $CsPbBr_3$ segment has been scaled down. Inset: PL image from which the spectrum is extracted.

Figure 6(c) shows Pb 4f spectra of a NW device at 0 V, which has already been under electrical operation before, with +1 V applied for one hour under X-ray exposure (at a longer integration time of 75 ms and a smaller step size of 40 nm). Therefore, the device is not in a pristine state anymore. The spectra of the device in the pristine state are shown in Figure S4(a), where the oxidized component is significantly stronger. The intensity of the reduced Pb^{+0} component is notably faint before a bias is applied.

At the pristine status of the double heterojunction device, as shown in Figure S4(a), there is a shift in the center part of the NW (anion exchanged) to a higher binding energy, which agrees with the proposed band-bending structure, illustrated in Figure S4(c).

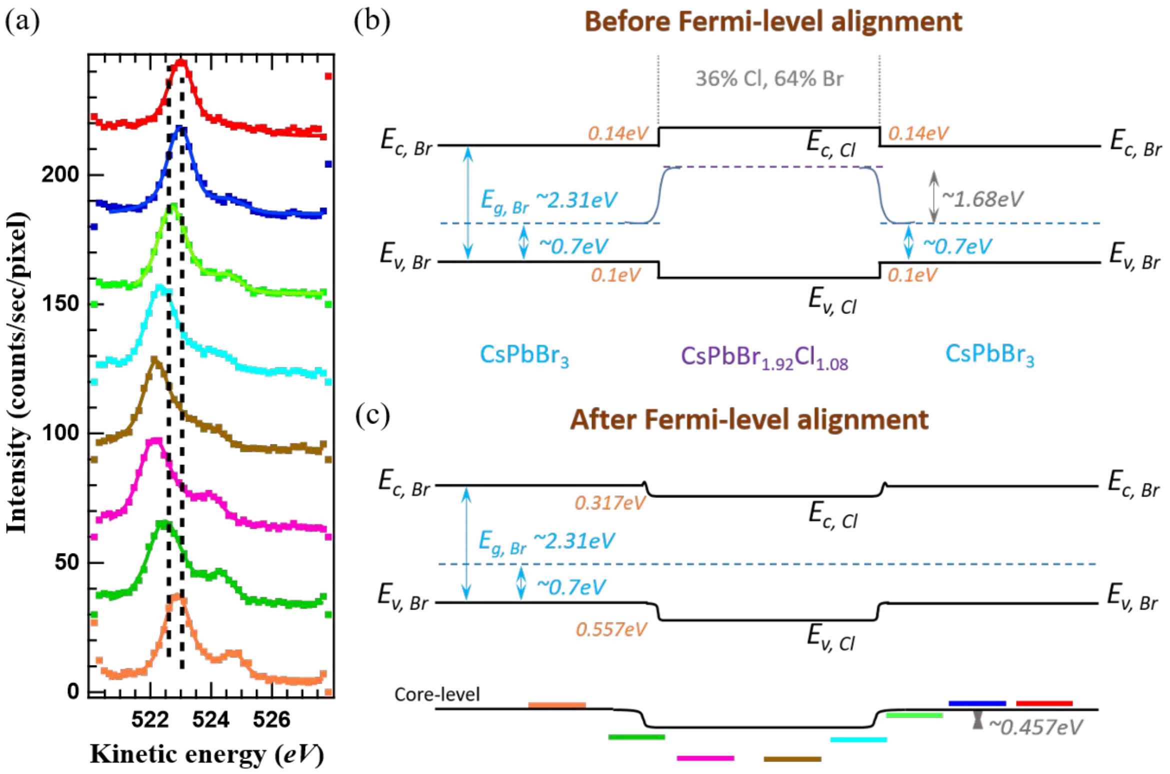

Figure S4. (a) Pb 4f spectra along the double-heterojunction NW device at the pristine stage. Band structure of the heterojunction NW device with $CsPbBr_3$ and $CsPbBr_{1.92}Cl_{1.08}$ segments (b) before and (c) after Fermi-level alignment, with band bending at the interfaces and the expected core-level band.

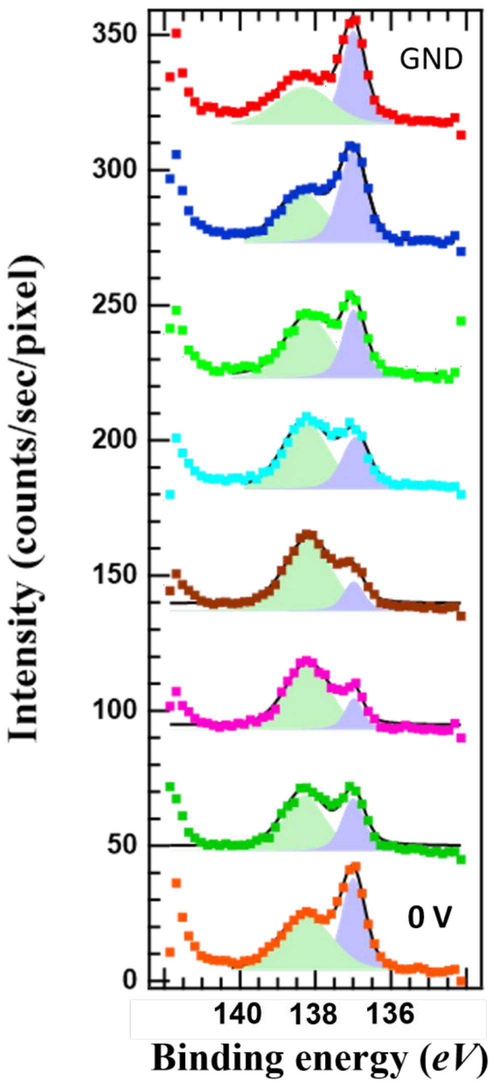

Figure S5. Pb $4f_{7/2}$ spectra fitting of metallic state (light violet) and oxidation state (light grass green) along the double-heterojunction NW device at 0 V, as shown in Figure 6(c). The dots are

the raw data; the black solid waves are the fitting curves; the light violet waves are the metallic state Pb^0; the light grass green waves are the oxidation state Pb^{2+}.

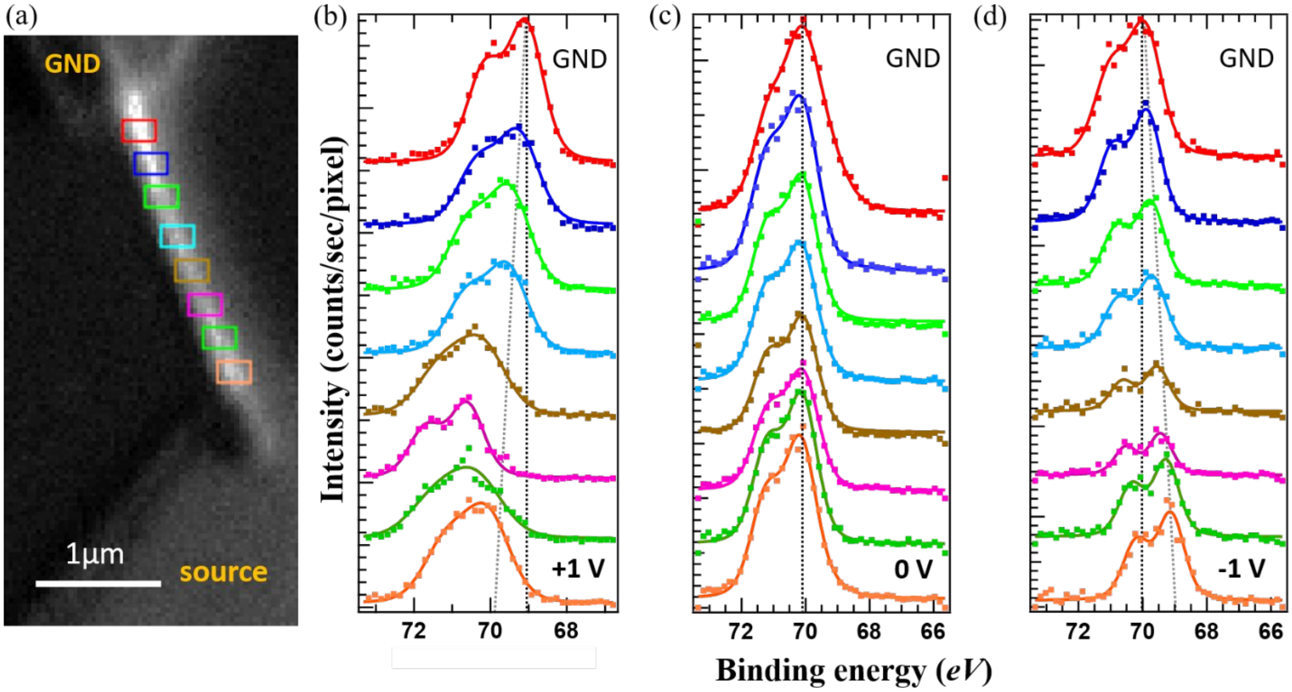

Figure S6. Br 3d core-level data from the same double heterostructure NW device as shown in Figures 6, S3, and S4 upon *operando* measurements within ± 1V, measured in parallel with the Pb 4f data displayed in Figure 6. (a) SPEM image, obtained at a binding energy range of 70 ± 3.5 eV and with an image step size of 40 nm. The positions of eight ROIs along the NW are indicated, as are the biased source electrode and the grounded ("GND") drain electrode. (b-d) Br 3d spectra, extracted at the different ROIs marked in (a) from SPEM images obtained under (b) +1 V, (c) 0 V, and (d) -1 V applied to the source electrode. The black dotted lines mark the binding energy

peak position of the Br $3d_{5/2}$ component next to the ground electrode, and the gray dotted lines indicate the trend of linear bias drop. Spectra of (c) and (d) are obtained at a binding energy range of 69.5 ± 4 eV, while that of (b) are obtained at the range of 70 ± 3.5 eV.
